# The Role of Large Language Models (LLMs) in Hepato-Pancreato-Biliary Surgery: Opportunities and Challenges

**DOI:** 10.7759/cureus.85979

**Published:** 2025-06-14

**Authors:** Guangbin Chen, Yanguang Sha, Zhilin Wang, Ke Wang, LongJiang Chen

**Affiliations:** 1 Department of Hepatobiliary Surgery, The Second People's Hospital of Wuhu, Wuhu Hospital Affiliated to East China Normal University, Wuhu, CHN; 2 Graduate School, Wannan Medical College, Wuhu, CHN; 3 Department of Hepatobiliary Surgery, Affiliated Yijishan Hospital of Wannan Medical College, Wuhu, CHN

**Keywords:** artificial intelligence, chatgpt, clinical decision support, hpb surgery, large language models, pancreatic surgery, surgical innovation

## Abstract

The integration of large language models (LLMs) into healthcare represents a transformative development in modern medicine, with hepato-pancreato-biliary (HPB) surgery positioned at the forefront of this technological revolution. This editorial examines the multifaceted role of LLMs, including ChatGPT, Claude, and Gemini, in HPB surgical practice, analyzing both their unprecedented opportunities and significant challenges. LLMs demonstrate remarkable potential across multiple domains of HPB surgery, including clinical documentation automation, enhanced surgical decision-making through complex patient data analysis, improved preoperative planning with radiological interpretation, real-time intraoperative decision support, and optimized postoperative patient monitoring with personalized follow-up recommendations. However, significant challenges accompany these opportunities, including data privacy and security concerns, accuracy and reliability issues, particularly regarding artificial intelligence (AI) "hallucinations," ethical considerations involving accountability and algorithmic bias, potential overreliance in surgical education that may compromise critical thinking development, and the risk of exacerbating healthcare disparities in resource-limited settings. Future directions include advancing multimodal learning capabilities, developing specialized HPB-focused LLMs, fostering collaborative development between surgeons and AI researchers, and establishing robust ethical frameworks with regulatory guidelines for safe implementation. The integration of LLMs in HPB surgery represents a paradigm shift requiring thoughtful navigation of opportunities and challenges, with the potential to transform surgical practice while maintaining the highest standards of patient safety and care quality through collaborative efforts among surgeons, AI researchers, ethicists, and policymakers.

## Editorial

The integration of artificial intelligence (AI) into healthcare has transformed various medical fields, with hepato-pancreato-biliary (HPB) surgery being no exception. Large language models (LLMs), such as ChatGPT (OpenAI), Claude (Anthropic), and Gemini (Google), have emerged as pivotal tools that offer unprecedented opportunities to enhance surgical practice [1,2]. LLMs are advanced AI systems trained on vast amounts of text data, capable of understanding and generating humanlike text across a wide range of topics and tasks. As experienced surgeons and clinicians, we critically examine the role of LLMs in HPB surgery, highlighting both their transformative potential and the challenges they present. While LLMs offer promising applications, their implementation is accompanied by significant considerations that must be carefully managed. This editorial provides a comprehensive overview of LLM applications in HPB surgery, discusses associated challenges, and offers insights into future directions for their implementation.

Applications of LLMs in HPB surgery

Clinical Documentation and Administrative Efficiency

LLMs have demonstrated remarkable capabilities in automating clinical documentation, which is a critical yet time-consuming aspect of surgical practice [3]. Studies show that doctors spend 7.7% of their working time on administrative activities [4]. In HPB surgery, where complex procedures and detailed patient histories are commonplace, LLMs can efficiently generate preoperative assessments, operative notes, and discharge summaries. This not only saves valuable time for surgeons but also enhances the quality and consistency of medical records, potentially improving patient care and facilitating research.

Augmenting Surgical Decision-Making

One of the most significant applications of LLMs in HPB surgery is their potential to support surgical decision-making. By analyzing patient data, including medical histories, imaging studies, and laboratory results, these models can provide surgeons with comprehensive insights into treatment strategies [5,6]. For instance, in cases of pancreatic cancer or complex liver resections, LLMs can assist in predicting postoperative outcomes, helping surgeons tailor their approach to individual patients [7,8]. Such predictive capabilities can help surgeons tailor their approaches to individual patients, particularly in complex scenarios such as borderline resectable pancreatic cancer or patients with significant comorbidities.

Enhanced Preoperative Planning

Preoperative planning, a crucial step in HPB surgery, can be significantly improved through LLM integration. When combined with imaging processing systems, these models can interpret complex radiological data and help in creating detailed 3D visualizations of anatomical structures [9]. This capability is particularly valuable in liver surgery, where understanding the intricate vascular and biliary anatomy is paramount for successful resection. LLM is used in a variety of application scenarios in the medical and healthcare fields, including clinical decision support and administrative task assistance [5]. In addition, LLM can provide guidance in the perioperative period, for example, by recommending advice on thromboembolic prophylaxis [10].

Intraoperative Guidance

Intraoperatively, LLMs have the potential to serve as real-time decision-support systems. By integrating with surgical navigation systems and processing live data, LLMs can provide instant guidance on critical decisions [11]. For example, during complex liver resections, an LLM could analyze intraoperative ultrasound findings and provide recommendations regarding the extent of resection or the management of unexpected findings.

Postoperative Care Optimization

Postoperative care and follow-up, which are crucial components of HPB surgery, can benefit significantly from LLM integration. These models can assist in monitoring patient recovery, predicting complications, and providing personalized recommendations for follow-up care [5,6]. This early detection capability is particularly beneficial in managing the complex postoperative course often observed in major HPB surgeries, potentially reducing length of stay and readmission rates.

Quality Improvement and Research

Furthermore, LLMs show promise in analyzing surgical outcome data to improve quality improvement initiatives in HPB surgery [5]. By processing large datasets of patient outcomes, surgical techniques, and perioperative management strategies, LLMs can identify patterns and trends that may not be apparent via traditional statistical analyses [5,12]. Such insights could lead to the development of more effective clinical pathways and personalized treatment protocols.

Challenges of LLMs in HPB surgery

Data Privacy and Security

Despite their promise, the implementation of LLMs in HPB surgery faces significant challenges. Foremost among these issues is data privacy and security [5-7]. The sensitive nature of patient information in surgical contexts necessitates robust safeguards to ensure confidentiality and compliance with regulatory standards, such as the Health Insurance Portability and Accountability Act (HIPAA) [13]. Implementation requires advanced data encryption, secure storage solutions, and strict access controls.

Accuracy and Reliability Concerns

The accuracy and reliability of LLMs in clinical decision-making remain critical concerns. While these models have shown impressive capabilities, their propensity for "hallucinations," generating plausible but incorrect information, poses significant risks in the high-stakes environment of HPB surgery [5-7]. LLMs can produce errors and misleading information when dealing with technical problems, especially if they are trained with only small datasets [14]. This highlights the need for rigorous validation protocols and continuous monitoring of LLM outputs, particularly when used for clinical decision support.

Ethical Considerations and Accountability

Ethical considerations regarding the use of AI in surgical decision-making are paramount. Questions of accountability, informed consent, and the potential for algorithmic bias must be addressed before widespread adoption of LLMs in surgical practice can occur [5,6,13,15]. Some LLMs demonstrated biases in treatment recommendations based on demographic factors, potentially exacerbating existing healthcare disparities [16]. The development of clear guidelines and ethical frameworks for the use of LLMs in surgery is an urgent priority in the medical community.

Integration With Surgical Education

The integration of LLMs into surgical education and training presents both opportunities and challenges. While these models can provide trainees with access to vast amounts of knowledge and simulated scenarios, there is a risk of overreliance on AI, potentially compromising the development of critical thinking skills essential for surgeons [5,17,18]. A balance must be struck between leveraging LLMs as educational tools and ensuring that trainees develop independent clinical reasoning abilities. Structured educational frameworks that incorporate LLMs as supplements to, rather than replacements for, traditional mentorship are essential [19].

Healthcare Disparities

Moreover, the potential of LLMs to exacerbate existing healthcare disparities, particularly in resource-limited settings, must be carefully considered [20]. The implementation of AI technologies requires substantial infrastructure and expertise, which may not be equally available across healthcare systems [21,22]. This could lead to a widening gap in the quality of care between well-resourced and underresourced institutions. Strategies to address this challenge include developing lightweight LLM applications that can function with limited computational resources and establishing international collaborations to ensure equitable access to these technologies [23].

Future directions

Multimodal Learning and Specialized Models

The future of LLMs in HPB surgery holds immense promise. Advancements in multimodal learning, which combines text-based knowledge with image and video processing, could lead to more sophisticated surgical planning and intraoperative guidance systems [11]. The development of specialized LLMs trained on HPB-specific datasets could further increase their utility in complex surgical subspecialties [5]. These specialized models, trained on annotated operative reports, imaging studies, and outcome data specific to HPB procedures, have demonstrated superior performance compared to general-purpose LLMs in preliminary validation studies.

Collaborative Development

Collaboration between HPB surgeons and AI researchers is crucial in developing tailored LLMs for surgical applications. These partnerships could focus on creating models that incorporate domain-specific knowledge, surgical techniques, and outcome data unique to HPB procedures [24]. The establishment of international consortia, such as the Surgical AI Collaborative announced in 2024, facilitates the sharing of anonymized datasets and technical expertise across institutions [25]. Such collaborations could also address the need for standardized protocols for validating and implementing LLMs in clinical practice, ensuring their reliability and effectiveness across different healthcare settings.

Regulatory Frameworks and Implementation Guidelines

The development of robust ethical frameworks and regulatory guidelines for the use of LLMs in surgery is imperative to ensure their safe and responsible integration into clinical practice [13]. Ongoing dialogue among surgeons, AI developers, ethicists, and regulatory bodies will be crucial for navigating the complex landscape of AI in healthcare. Several professional societies, including the Society for Surgery of the Alimentary Tract and the International Hepato-Pancreato-Biliary Association, have recently established task forces to develop specialty-specific guidelines for AI implementation.

Validation and Quality Control

Implementation of rigorous quality control measures for training data and model validation is essential for ensuring the safety and efficacy of LLMs in HPB surgery. This includes the development of standardized benchmarks for assessing model performance, regular auditing of model outputs, and continuous monitoring for bias or errors [26]. The recent proposal by the American College of Surgeons for an AI certification program represents an important step toward establishing standardized validation protocols for surgical AI applications [27].

Conclusion

The integration of LLMs in HPB surgery represents a significant advancement in the convergence of AI and medicine. Although these models offer substantial benefits, from streamlining documentation to enhancing surgical decision-making, they also present challenges that must be addressed to realize their full potential. Specific recommendations for the HPB surgical community include fostering collaboration with AI researchers, developing specialized LLMs trained on procedure-specific datasets, establishing standardized validation protocols, and contributing to the development of ethical guidelines for AI implementation.

As we navigate this new frontier, collaboration among surgeons, AI researchers, ethicists, and policymakers will be crucial in harnessing the potential of LLMs while safeguarding patient safety and maintaining the highest standards of surgical care. With thoughtful implementation and ongoing refinement, LLMs have the potential to transform HPB surgery, ultimately improving outcomes for our patients.
